# Supplementary material for: Biocontrol Effect of Clonostachys rosea on Fusarium graminearum Infection and Mycotoxin Detoxification in Oat (Avena sativa)
Source: Plants (Basel). 2023 Jan 21;12(3):500. doi: 10.3390/plants12030500 (PMC9918947; doi:10.3390/plants12030500)
Supplement: Supplementary file 1 [file plants-12-00500-s001.zip › Supplementary Figure S2. F.gram symptoms in oat.pdf]

Supplementary Figure S2. Symptoms *of F.graminearum* infection in mature oat

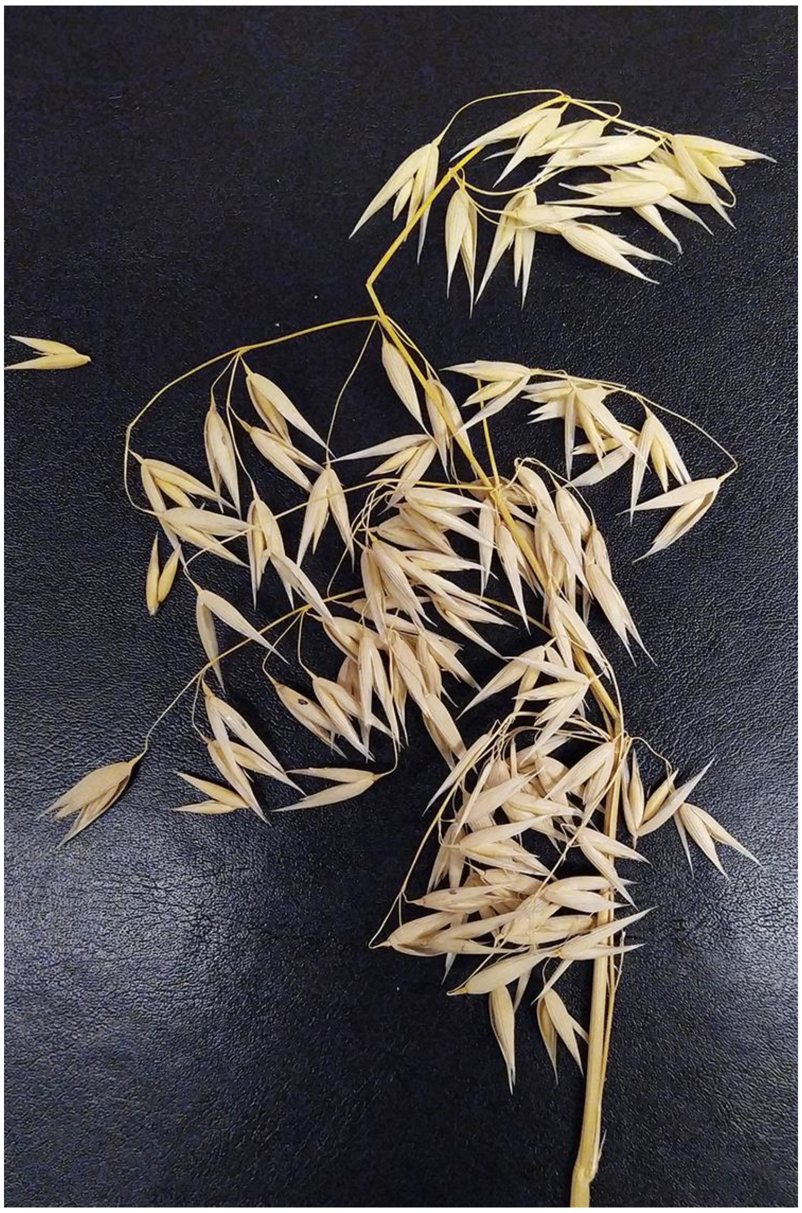

Image 1. An oat panicle

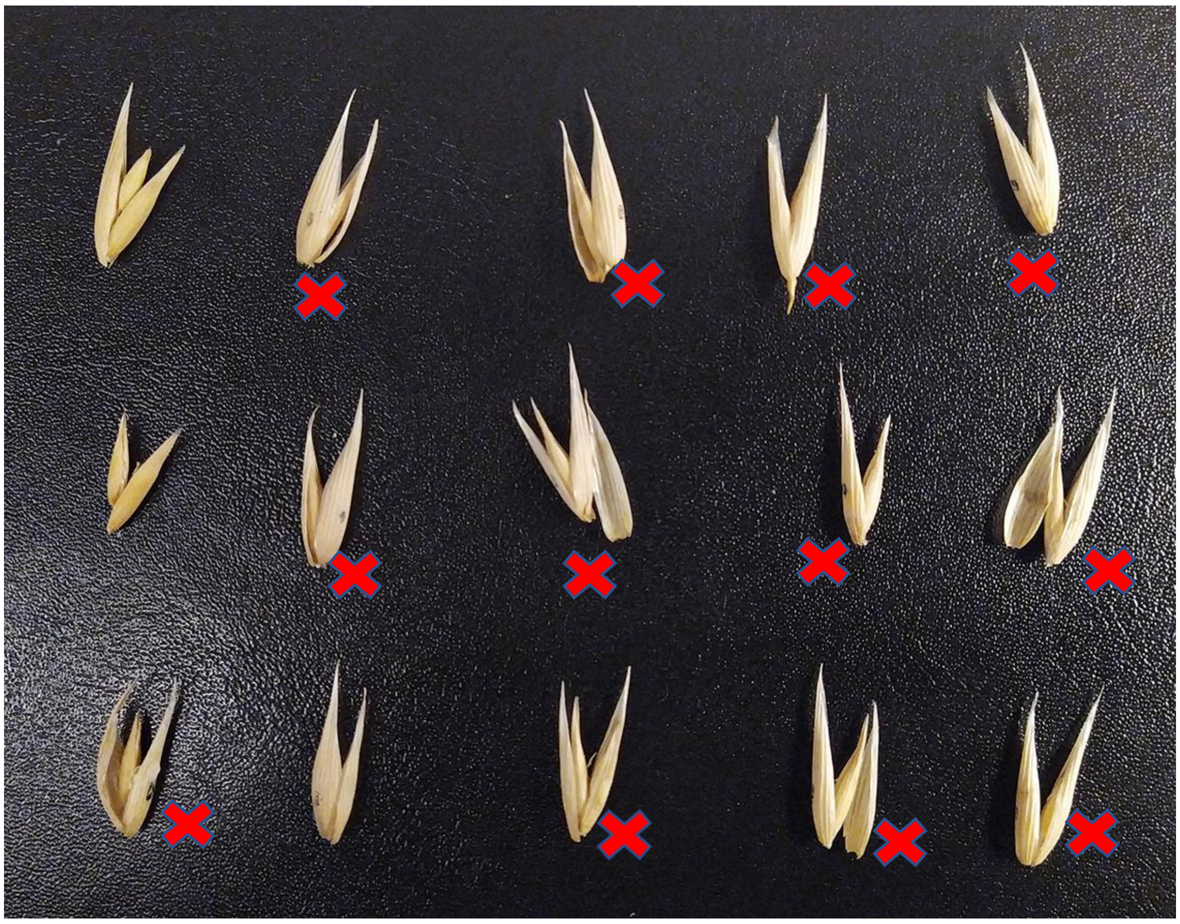

Image 2. Separate spikelets. Marked spikelets contain damaged kernels (one or two), which can be seen only when de-hulled (see image 3) .

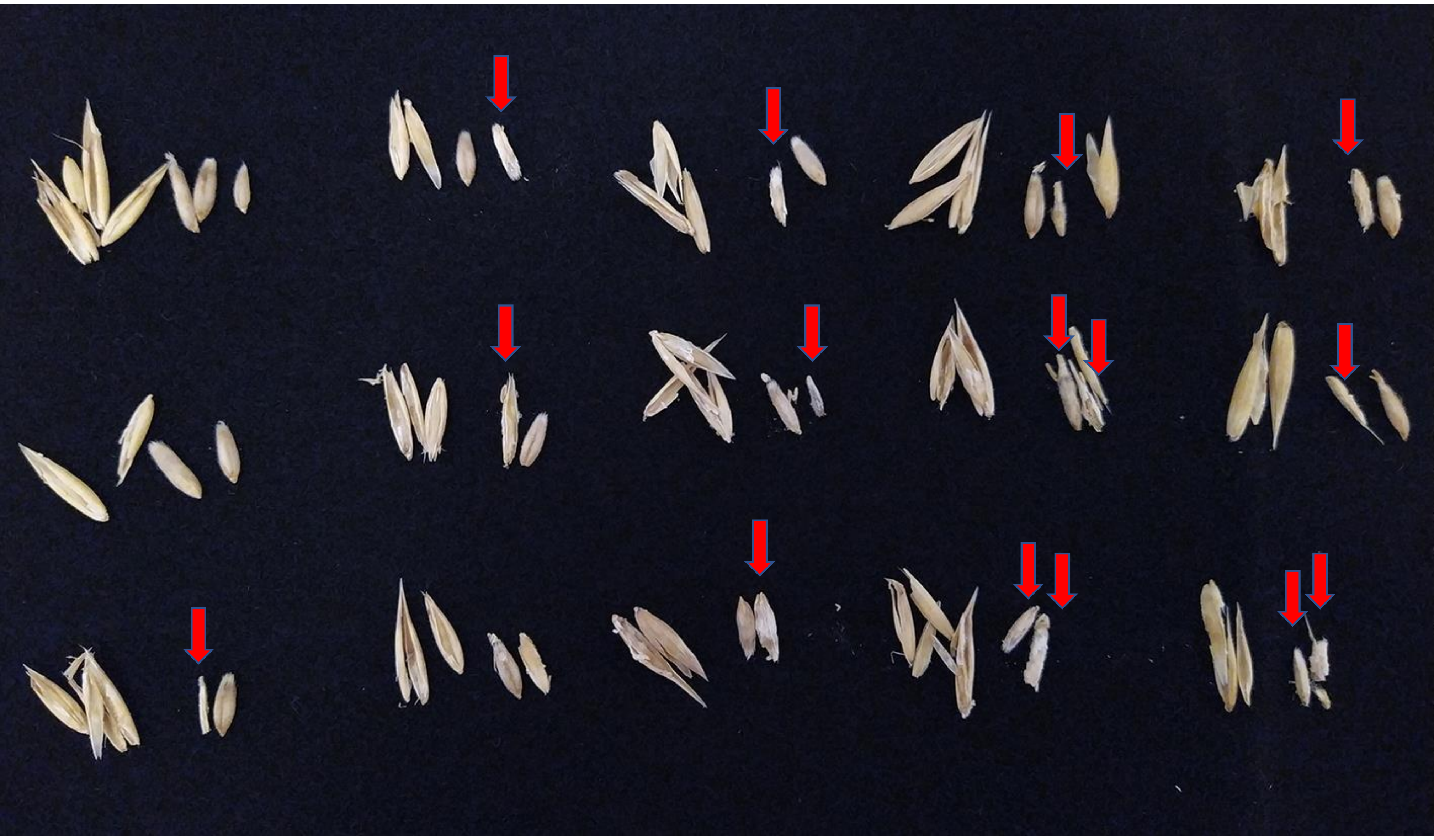

3. De-hulled oat spikelts: mature oat kernels with corresponding hulls (same order as on image 2.)
