# Supplementary material for: Biocontrol Effect of Clonostachys rosea on Fusarium graminearum Infection and Mycotoxin Detoxification in Oat (Avena sativa)
Source: Plants (Basel). 2023 Jan 21;12(3):500. doi: 10.3390/plants12030500 (PMC9918947; doi:10.3390/plants12030500)
Supplement: Supplementary file 1 [file plants-12-00500-s001.zip › Supplementary Table S1.pdf]

Supplementary Table S1. **Primers and probes used in this work:**

gene	gene designation in cv.Sang genome	primer/probe name	sequence
Tubulin-α	AVESA.00010b.r2.7CG0700360.1	Tub-a forward<br>Tub-a reverse<br>Tub-a probe	GAG GTG GAA CTG GCT CTG<br>GAC ACT GTT GTA TGG CTC AAC<br>/5HEX/TCA CCT CAG /ZEN/GTC TCC ACC TCT GTT /3IABkFQ/
AsUGT1	AVESA.00010b.r2.6AG1068650.1	AsUGT1 forward<br>AsUGT1 reverse<br>AsUGT1 probe	GTG GAT GGA ACT CAA CAT TGG AG<br>GGT CAG CCC AAT CTG GAA TAC<br>/56-FAM/CCG TTG CTA /ZEN/GCG GTG TAC CTC TTG /3IABkFQ/
AsUGT2	AVESA.00010b.r2.6AG1068570.1	AsUGT2 forward<br>AsUGT2 reverse<br>AsUGT probe	GAC CAA CCC ACC ATC TCA AA<br>CCC ATT CAT CAC CTC TCT AAT ACA A<br>/56-FAM/TTT CTC TCT /ZEN/TCC GCA CTC GCA CAC /3IABkFQ/
PR1	AVESA.00010b.r2.7AG1201740.1<br>AVESA.00010b.r2.UnG1401040.1	PR1 forward<br>PR1 reverse<br>PR1 probe	TGC AAG CTC CAG CAC TC<br>GCT GTT GGA CCC ATA GTT GTA<br>/56-FAM/AAC CTGT GGG /ZEN/TCA GCG AGA AGA AT/3IABkFQ/
PR3	AVESA.00010b.r2.1DG0178620<br>AVESA.00010b.r2.1AG0060060	PR3 forward<br>PR3 reverse<br>PR3 probe	GGC TAC TGC TTC AAG GAA GA<br>CCA GCT GGT AGT TGG ACT G<br>/56-FAM/ACC AAG GAC /ZEN/GTG GAC CAA TCC AAT/3IABkFQ/
PR4	AVESA.00010b.r2.4AG0575210<br>AVESA.00010b.r2.4DG0719670	PR4 forward<br>PR4 reverse<br>PR4 probe	ACT GGA CAC CGT CTT CT<br>CAG TCG ACG AAC TTG TAG TTG A<br>/56-FAM/CAA GAT CGA /ZEN/CAC CAA CGG ACA GGG/3IABkFQ/
PR5	AVESA.00010b.r2.5DG0992430.1<br>AVESA.00010b.r2.5AG0816180.1	PR5 forward<br>PR5 reverse<br>PR5 probe	CGA ACT ACT CCC AGT TCT TCA A<br>CAGAGCACGATCTGGTAGTTT<br>/56-FAM/ACG ACC AGA /ZEN/CAA GCA CAT TCA CCT/3IABkFQ/
WRKY23-like	AVESA.00010b.r2.7DG1345990.1<br>AVESA.00010b.r2.2AG0259040.1<br>AVESA.00010b.r2.6CG1146320.1	WRKY23 forward<br>WRKY23 reverse<br>WRKY23 probe	CAG AAG GTG ACC AAG GAC AA<br>CGC TGC ACC TTC TTC TTC A<br>/56-FAM/TAC TTC CGG /ZEN/TGC TCC TTC GC/3IABkFQ/
WRKY70-like	AVESA.00010b.r2.1DG0191960.1<br>AVESA.00010b.r2.1AG0073190.1<br>AVESA.00010b.r2.1CG0105620.1	WRKY70 forward<br>WRKY70 reverse<br>WRKY70probe	CGG CGG GAA GAG AAA GTC<br>GTC GTC CAA GCT CTT CAA GT<br>/56-FAM/AGA AAG ACC/ZEN/ CAG CAA TCG TCC GTT/3IABkFQ/
