# Supplementary material for: Biocontrol Effect of Clonostachys rosea on Fusarium graminearum Infection and Mycotoxin Detoxification in Oat (Avena sativa)
Source: Plants (Basel). 2023 Jan 21;12(3):500. doi: 10.3390/plants12030500 (PMC9918947; doi:10.3390/plants12030500)
Supplement: Supplementary file 1 [file plants-12-00500-s001.zip › Supplementary Table S2.pdf]

Supplementary Table S2**. MRM transitions and retention times.**

Name	Short Name	RT (min)	Precursor	Product	CE
Deoxynivalenol-3-glucoside	DON_3_Glu	2.10	459.20	297.1	10
Deoxynivalenol-3-glucoside	DON_3_Glu	2.10	459.20	249.1	20
Deoxynivalenol	DON	2.15	297.14	249.1	10
Deoxynivalenol	DON	2.15	297.14	231.0	20
Deoxynivalenol-13C15	DON-IS	2.15	312.00	263.0	15
Deoxynivalenol-13C15	DON-IS	2.15	312.00	245.1	20
Caffeine-13C3	Caffein-IS	2.20	198.10	140.0	20
3-Acetyl-Deoxynovalenol-13C17	3_AcDON-IS	2.55	356.00	245.0	15
Zearalenone	ZEA	3.40	317.10	273.1	18
Zearalenone	ZEA	3.40	317.10	175.1	26
Zearalenone-13C18	ZEA-IS	3.40	334.80	183.8	26
