# Supplementary figures and images for: Biocontrol Effect of Clonostachys rosea on Fusarium graminearum Infection and Mycotoxin Detoxification in Oat (Avena sativa)
Source: Plants (Basel). 2023 Jan 21;12(3):500. doi: 10.3390/plants12030500 (PMC9918947; doi:10.3390/plants12030500)

Supplementary Figure S1. Additional DON metabolites (expressed as “area/mg”)

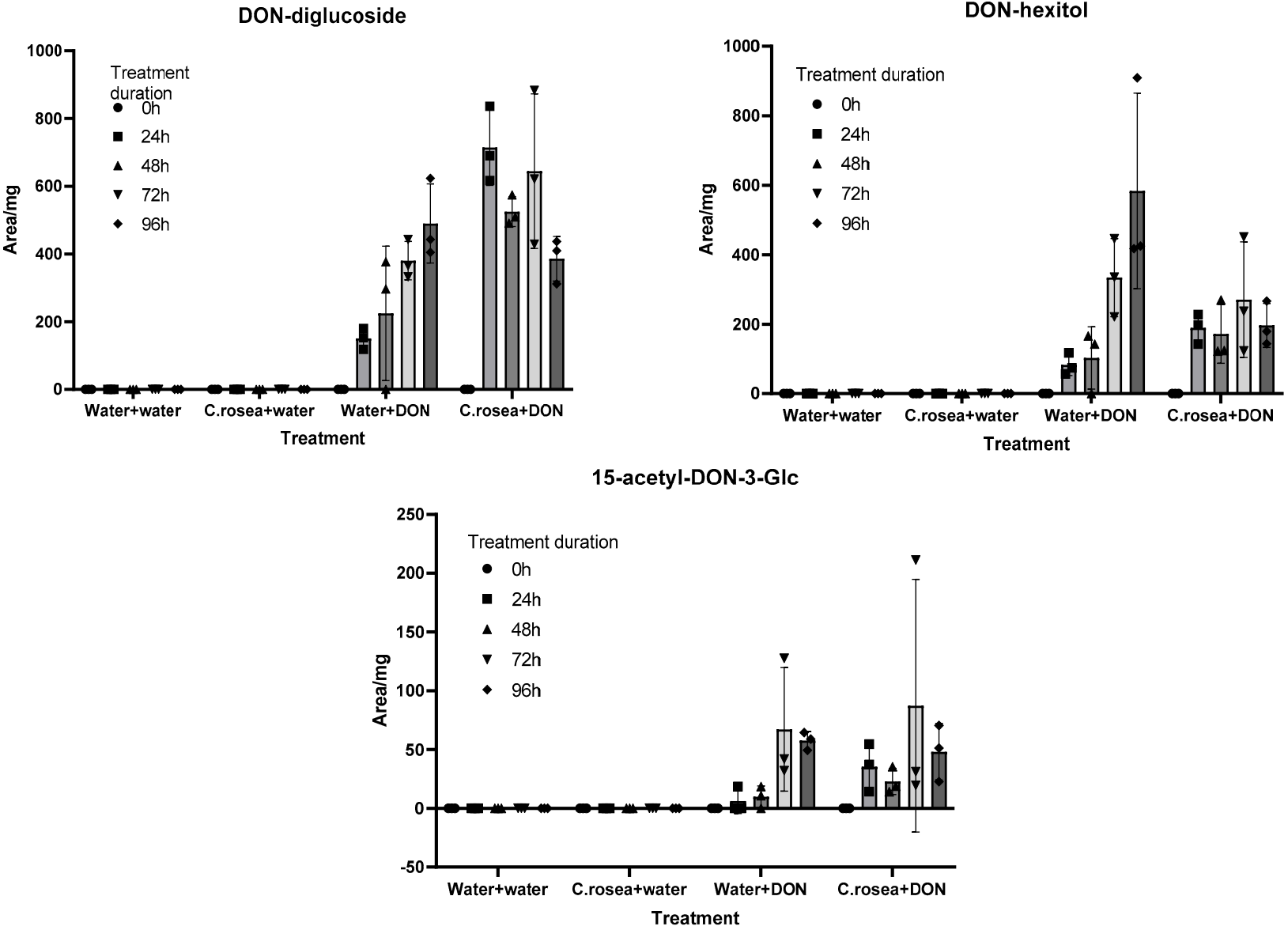

Supplement: Supplementary file 1 [file plants-12-00500-s001.zip › Supplementary Figure S1.pdf]
